# Emergency department characteristics and capabilities in Bogotá, Colombia

**DOI:** 10.1186/s12245-015-0079-y

**Published:** 2015-08-08

**Authors:** Yury Bustos, Jenny Castro, Leana S. Wen, Ashley F. Sullivan, Dinah K. Chen, Carlos A. Camargo

**Affiliations:** Department of Emergency Medicine, Universidad del Rosario, Carrera 24 N° 63C-69, Bogotá, Colombia; Department of Emergency Medicine, Universidad del Rosario/Hospital Universitario Mayor-Méderi, Bogotá, Colombia; Department of Emergency Medicine, Georgetown Washington University School of Medicine, Washington, DC USA; Department of Emergency Medicine, Massachusetts General Hospital, Boston, MA USA

**Keywords:** International emergency medicine, Bogotá, Colombia, Emergency department classification, Health policy, Emergency department crowding

## Abstract

**Background:**

Emergency departments (EDs) are a critical, yet heterogeneous, part of international emergency care. The National ED Inventories (NEDI) survey has been used in multiple countries as a standardized method to benchmark ED characteristics. We sought to describe the characteristics, resources, capabilities, and capacity of EDs in the densely populated capital city of Bogotá, Colombia.

**Methods:**

Bogotá EDs accessible to the general public 24/7 were surveyed using the 23-item NEDI survey used in several other countries (www.emnet-nedi.org). ED staff were asked about ED characteristics with reference to calendar year 2011.

**Results:**

Seventy EDs participated (82 % response). Most EDs (87 %) were located in hospitals, and 83 % were independent hospital departments. The median annual ED visit volume was approximately 50,000 visits. Approximately 90 % (95 % confidence interval (CI) 80–96 %) had a contiguous layout, with medical and surgical care provided in one area. Almost all EDs saw both adults and children (91 %), while 6 % saw only adults and 3 % saw only children. Availability of technological and consultant resources in EDs was variable. Nearly every ED had cardiac monitoring (99 %, 95 % CI 92–100 %), but less than half had a dedicated CT scanner (39 %, 95 % CI 28–52 %). While most EDs were able to treat trauma 24/7 (81 %, 95 % CI 69–89 %), few could manage oncological (22 %, 95 % CI 13–34 %) or dental (3 %, 95 % CI 0–11 %) emergencies 24/7. The typical ED length-of-stay was between 1 and 6 h in 59 % of EDs (95 % CI, 46–70 %), while most others reported that patients remained for >6 h (39 %). Almost half of respondents (46 %, 95 % CI 34–59 %) reported their ED was over capacity.

**Conclusions:**

Bogotá EDs have high annual visit volumes and long length-of-stay, and half are over capacity. To meet the emergency care needs of people in Bogotá and other large cities, Colombia should consider improving urban ED capacity and training more emergency medicine specialists capable of efficiently staffing its large and crowded EDs.

**Electronic supplementary material:**

The online version of this article (doi:10.1186/s12245-015-0079-y) contains supplementary material, which is available to authorized users.

## Background

With a population of 7.5 million people, Bogotá is not only Colombia’s largest city but one of the largest cities in South America [1]. The Colombian health system establishes health care as a fundamental right for all citizens. Law 100 of 1993 established the benefit of insurance plans, with an eventual goal of providing coverage to the entire population [2]. After several reforms to advance this and other goals, health insurance coverage in Colombia has reached approximately 90 %, and health expenditures account for approximately 7.6 % of the gross domestic product. Unfortunately, the supply of physicians is insufficient to meet the demand resulting from these health system changes. At present, for every 10,000 people, there are only 1.5 physicians [3].

These major health system changes directly affect emergency care, which, by its nature, requires immediate attention and exposes deficiencies of the system [4]. The description and study of emergency departments (EDs), as the basic unit for the emergency care system, can improve understanding of the strengths and weaknesses of Colombian emergency care, and enable comparison with other emergency care systems worldwide, as Colombia and many other countries search for solutions to common problems [5]. Data obtained in the National Emergency Department Inventory (NEDI) project has demonstrated already how heterogeneous EDs are within the same city and in different parts of a country [6, 7]. Our objective was to use the NEDI instrument to describe the fundamental characteristics of Bogotá EDs, with particular attention to available resources, capabilities, and capacity of these EDs.

## Methods

This was a cross-sectional descriptive study with web-based surveys administered to the physician-administrator at each Bogotá ED. Consistent with terminology used in NEDI-USA, an ED was defined as an emergency care facility that is open 24 h per day, 7 days per week. A list of EDs was obtained from the Centro Regulador de Urgencias y Emergencias de Bogotá and verified for completeness by two local emergency physicians (YB, JC). All eligible EDs were contacted and surveyed in 2012. The study was coordinated by the Emergency Medicine Network (EMNet) in Boston (www.emnet-nedi.org). This study was approved by the Ethics Committee of the Universidad del Rosario and determined to be exempt by the Partners Institutional Review Board.

The 23-item NEDI instrument was used. Participants were specifically asked in 2012 about ED characteristics with reference to calendar year 2011. Survey questions were drawn, in part, from a survey that has been administered in hundreds of US EDs [7]. Questions were subdivided into four categories: ED characteristics, patient experiences in the ED, capacity, and resources and capabilities. Before implementation, survey questions were reviewed by members of the EMNet Steering Committee and several on-site country coordinators of prior NEDI international surveys [6, 8–12]. The survey was translated into Spanish by a professional interpreter service and independently checked for accuracy by two bilingual emergency physicians (Additional file 1).

Because many Bogotá ED directors reported uncertainty about their estimate of annual ED visit volume (when completing the original 23-item survey), we then distributed a 5-item follow-up survey to check reliability of this key variable. The follow-up survey included the following variables: number of hospital beds, annual ED visit volume, percent of ED visits that led to hospital admission, number of hospital admissions, and percent of hospital admissions that were admitted through the ED.

Responses were entered into LimeSurvey (www.limesurvey.org) and downloaded into an Excel spreadsheet (Microsoft Corp., Redmond, WA). Responses received were maintained on a secure, password-protected server. Descriptive statistics, Wilcoxon rank-sum test, and Fisher’s exact test were calculated using Stata 11.0 (StataCorp, College Station, TX).

## Results

### General characteristics

**Fig. 1:**
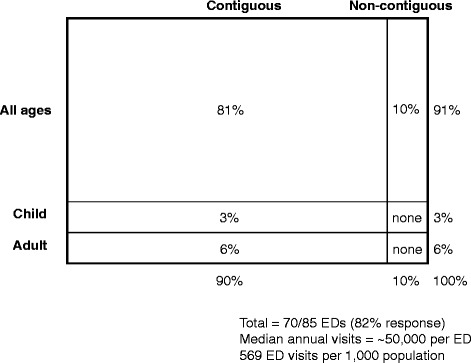
Snapshot of overall emergency department characteristics in Bogotá, Colombia

Among the 85 EDs in Bogotá at the time of survey administration, 54 % were located in private hospitals and 42 % were in teaching hospitals. Additional file 2 shows a map of the 85 EDs that were surveyed. Out of 85 EDs in Bogotá, 70 participated in the primary survey (82 % response rate). Respondent and non-respondent EDs did not differ with respect to ownership, metropolitan status, or academic status (data not shown). As shown in Table 1, 87 % were located in hospitals, and 83 % were independent hospital departments (i.e., not under the jurisdiction of medicine or surgery departments). Approximately 90 % had a contiguous layout with medical and surgical care provided in one area, with most (67 %) using triage to service (i.e., triage of patients to a specific emergency service, for example, medical vs surgical team). Almost all EDs saw both adults and children (91 %), while 6 % saw only adults and 3 % saw only children. The main survey (*n* = 70) yielded an estimate of 61,637 annual ED visits per year (median). Several respondents reported difficulty obtaining their ED visit volume data, and, for this reason, we performed a second survey of the participating sites to provide directors with more time and to assess the reliability of the original estimate. Among the 37 repeat respondents (53 % of the original 70 EDs), the median annual ED visit volume was 44,457 visits, with no significant difference in the median number of ED visits between surveys (*P* = 0.47). Comparing the two surveys, there was no significant difference in the number of hospitals beds, but there was a significant difference in the reported percentage of ED visits leading to admission and percentage of total hospital admissions through the ED (Table 2). Based on a population of 7,467,804 million (1), the estimated total number of ED visits in Bogotá yielded a population ED visit rate of between 627 (follow-up survey) and 772 (main survey) ED visits per 1000 people. Figure 1 provides a “snapshot” of the overall ED characteristics in Bogotá, using the approach used in several prior NEDI international surveys [6, 8–12].

**Table 1:** Characteristics of EDs in Bogotá, Colombia, based on the 70 sampled EDs

	Proportion or median	95 % confidence interval or interquartile range
General characteristics		
Hospital based	87 %	77–94 %
Independent department	83 %	72–91 %
Contiguous	90 %	81–96 %
Triage to service^a^	67 %	54–78 %
Annual ED visits (median)		
From main survey (*n* = 70)	61,637	29,900–114,159
From follow-up survey (*n* = 37)	44,457	19,920–129,215
ED beds (median)	19	11–37
Patient experiences in ED		
Percent of ED patients arriving by ambulance		
<20	63 %	50–74 %
Length-of-stay		
<1 h	3 %	0–10 %
1–6 h	59 %	46–70 %
>6 h	38 %	27–51 %
Percent of ED visit leading to admission^b^		
<20	3 %	0–14 %
20–39	84 %	68–94 %
40–59	13 %	5–29 %
60 or higher	0 %	0 %
Resources and capabilities		
Physician in ED 24/7	99 %	92–100 %
Dedicated CT scanner	39 %	28–52 %
Cardiac monitor	99 %	92–100 %
Mechanical ventilator	62 %	49–73 %
Respiratory isolation (negative-pressure room)	51 %	38–63 %
Computer system to collect clinical data	73 %	61–83 %
Internet access	80 %	69–89 %
Clinical laboratory open round-the-clock	83 %	72–91 %

*ED* emergency department

^a^Among contiguous EDs only

^b^Based on follow-up survey (*n* = 37)

**Table 2:** Selected characteristics of EDs in Bogotá, Colombia, based on the original survey (*n* = 70) and follow-up survey (*n* = 37)

	Original survey (*n* = 70)	Follow-up survey (*n* = 37)	*P* value
ED visits, median (IQR)	61,637 (30,000–107,317)	44,457 (19,920–129,215)	0.47
Hospital beds, median (IQR)	100 (16–204)	95 (33–186)	0.75
Percent of ED visits leading to admission			<0.001
<20	29	3	
20–39	37	84	
40–59	13	13	
60–79	10	0	
80+	11	0	
Percent of total hospital admissions admitted through the ED			0.003
<20	19	3	
20–39	10	31	
40–59	7	23	
60–79	15	9	
80+	49	34

### Patient experiences in the ED

Most respondents (63 %) answered that <20 % of their patient population arrived by ambulance. In 59 % of EDs, the average length-of-stay was between 1–6 h; in 38 %, the average length-of-stay was >6 h. Among EDs with an annual visit volume of 100,000 visits or more, 57 % reported the average length-of-stay as >6 h. Only 3 % of EDs reported that their patients typically stayed <1 h. Most EDs reported that 20–39 % of ED visits led to admission (Table 1).The ED appears to be a major route of hospital admission. Among the hospitals with inpatient beds available, 34 % of EDs (95 % confidence interval (CI), 19–52 %) reported that admissions from the ED constituted >80 % of all hospital admissions.

### Capacity

While 25 % of respondents (95 % CI, 15–37 %) considered their ED as operating at a good balance, 46 % (95 % CI, 34–59 %) considered it over capacity.

### Resources and capabilities

**Fig. 2:**
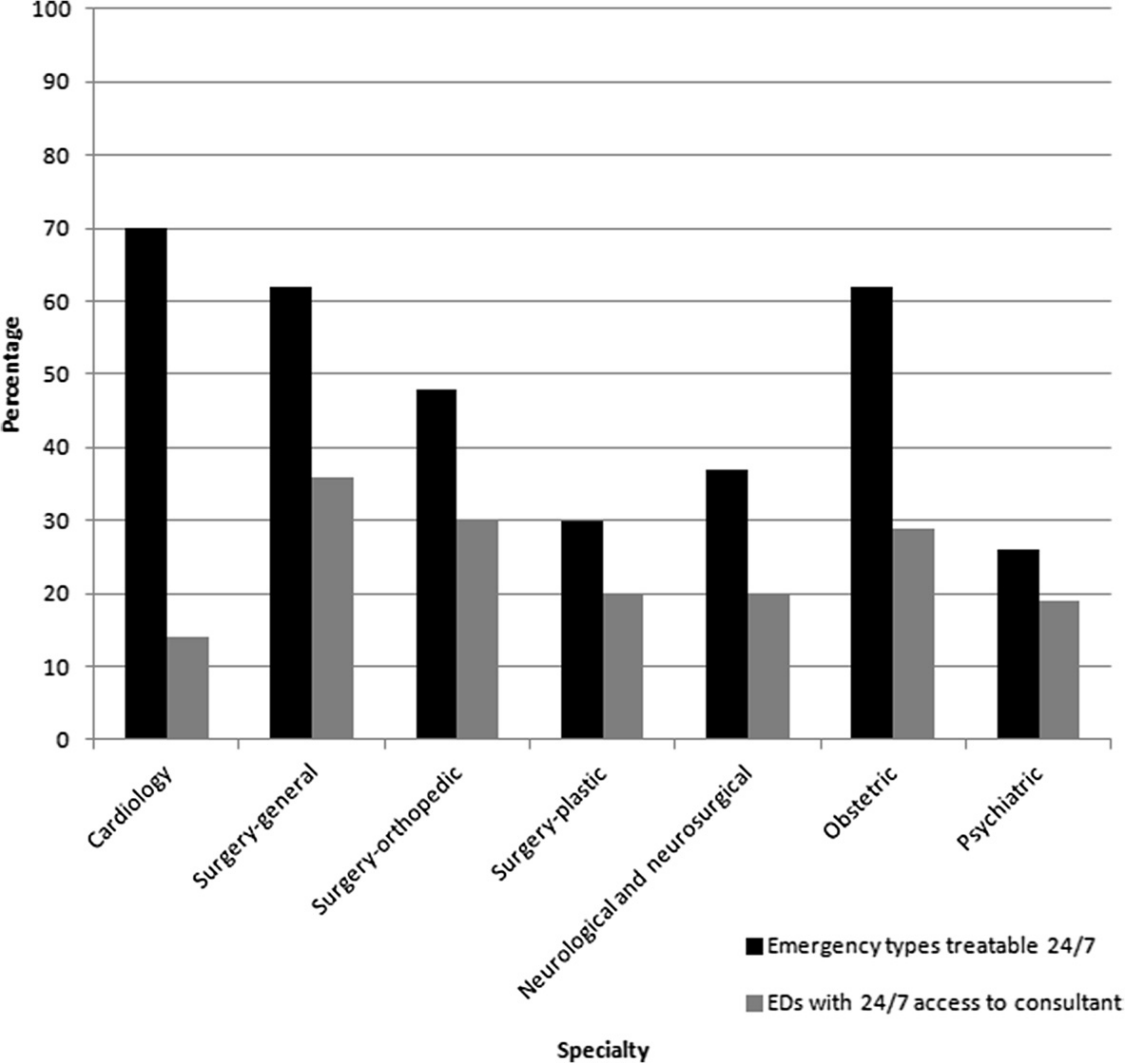
Association between emergencies identified as treatable and availability of consultants in the emergency departments, by type of emergency

The vast majority of EDs were staffed 24/7 by physicians (99 % CI, 92–100 %). Technological support was high, with most EDs having cardiac monitors (99 % CI, 92–100 %) and clinical laboratories (83 % CI, 72–91 %) open 24/7. However, internet and computer system access was variable (Table 1). Most emergency types could be treated 24/7 in sampled EDs (Table 3), with the notable exception of dental emergencies, which were treatable 24/7 in only 3 % of EDs (95 % CI, 0–11 %). The availability of consultants did not appear to correlate with the type of emergency that the EDs were capable of treating (Fig. 2).

**Table 3:** Emergency types identified as treatable in surveyed emergency departments in Bogotá, Colombia (*n* = 70, 82 % response rate)

Emergency type	Example of emergency	Percentage of EDs able to treat 24/7 (%)	95 % confidence interval (%)
Medical: cardiology	Arrhythmia, acute myocardial infarction	73	61–83
Medical: oncology	Fever and neutropenia	22	13–34
Medical: other	Urinary tract infection, acute asthma	85	74–93
Trauma	Motor vehicle crash, gunshot wound	81	69–89
Neurological and neurosurgical	Acute thromboembolic stroke, intracranial hemorrhage	37	25–49
Urological	Kidney stone	55	43–67
Obstetrical	Complications of pregnancy	63	51–75
Gynecological	Ruptured ovarian cyst, yeast infection	57	44–69
Ear, nose, throat	Severe epistaxis	41	29–53
Ophthalmological	Acute glaucoma, eye injury	25	15–37
Toxicological	Overdose, carbon monoxide poisoning	61	38–63
Psychiatric	Psychosis	26	16–39
Dental	Tooth extraction	3	0–11
Surgical: oral maxillofacial	Jaw fracture, oral abscess	29	18–41
Surgical: plastic	Severe lip laceration	30	20–43
Surgical: hand	Tendon injury	30	20–43
Surgical: orthopedic	Long bone fractures	47	35–60
Surgical: general	Acute appendicitis, pneumothorax	60	48–72

*ED* emergency department

## Discussion

The NEDI-Bogotá survey was successfully implemented, with >80 % response rate among all EDs. In some ways, the Bogotá EDs are quite similar to those in the United States (US) [6, 13]. For example, the vast majority of EDs have a contiguous area with assessment of medical and surgical emergencies in the same area. In general, EDs are independent departments and most have essential technological resources, such as cardiac monitors and 24/7 clinical laboratories.

However, the survey also identified several important differences between typical EDs in Bogotá and the US. The population rate of ED visits in Bogotá (~600 per 1000 people) is much larger than that seen in the US and several NEDI international surveys worldwide (Table 4). The reasons for this very high population rate are unclear, and it cannot be explained by high population density alone since this also would apply to other NEDI sites, such as Beijing [8]. Another factor that is frequently cited as a contributor to high ED visit volume is a relatively weak primary care system, but this situation would not necessarily apply to Colombia and does not provide a clear pattern when reviewing results from other cities/countries that have completed NEDI surveys [8–12, 14], particularly our report on Abuja, Nigeria [10].

**Table 4:** Summary of international National Emergency Department Inventories (NEDI), including the current study in Bogotá, Colombia

Location (reference)	City vs country	Survey response	Contiguous ED for all ages (%)	Median annual ED visits	ED visits per 1000 population
Abuja, Nigeria [10]	City	24/29 = 83 %	92	1500	54
Beijing, China [8]	City	36/41 = 88 %	36	80,000	167
Singapore [12]	City/country	14/14 = 100 %	86	39,450	197
Slovenia [9]	Country	55/68 = 81 %	90	6100	207
Denmark [11]	Country	28/34 = 82 %	39	32,000	196
Switzerland [14]	Country	122/138 = 88 %	na	8806	214
USA [7]	Country	>80 % (multiple)	na	20,351	437
Bogotá, Colombia	City	70/85 = 82 %	90	~50,000	569

*ED* emergency department, *na* not applicable

Prior work on Colombian EDs is very limited. A survey of 560 patients with triage classifications that indicated a low acuity condition or one that could be treated in an outpatient clinic, from 28 Colombian EDs, examined reasons for visiting the ED instead of an outpatient clinic [15]. The most commonly reported reason (23 %) was that the person visited the ED because of a delay in his or her outpatient appointment; 15 % said that making an outpatient appointment was too complicated, 15 % of patients reported visiting an outpatient clinic without resolution of the problem, 13 % stated a preference for the quality of emergency care, and 11 % reported other reasons. Although it is tempting to generalize these results to the Bogotá EDs in the present study, further examination of these earlier data suggests caution. For example, although the six Bogotá EDs together contributed 45 % of all ED visits in the 28-center dataset [15], these same six EDs correspond to only 7 % of the 85 EDs that we identified in Bogotá [15, 16]. While it is possible that they accurately represent at least some aspects of emergency care in Bogotá, it is likely that a focus on larger, more academically inclined EDs introduces a systematic error (bias) that complicates generalizations about the state of ED and emergency care in Bogotá, Colombia.

Other insights from our survey include the fact that most Bogotá EDs serve both children and adults, which is consistent with the practice observed in most prior NEDI international surveys, with the notable exception of Beijing, where 55 % of EDs were dedicated to adults only [8]. Bogotá has a population one third the size of Beijing (20.6 million), but Bogotá has twice as many EDs, which suggests that Beijing probably has a larger percentage of institutions of increased complexity and quite different dynamics of health care. These cross-country comparisons merit further investigation.

Furthermore, we found that most Bogotá EDs were able to care for many different types of emergencies, except for a notable lack of capability in the care of psychiatric, oncological, ophthalmological, and dental emergencies. These findings probably are explained by the fact that most Colombian doctors working in the ED are not specialized in emergency care or any other particular area; they are generalist physicians without particular training or expertise in the identified areas. Yet, the types of emergencies surveyed are all within the scope of practice of trained EM practitioners [17]. In settings limited by workforce constraints, there may be additional reason to train EM physicians with a core set of skills to address initial management of all common emergencies [8, 10].

Regarding the ED length-of-stay, 38 % of EDs reported typical stays of over 6 h and 59 % between 1 and 6 h. In addition, about half of the EDs surveyed reported that they were over capacity. We believe this is largely due to the fact that most of the EDs are primarily primary care centers. In other words, many ED patients present with issues of low complexity, so ED stays are shorter in duration. This is in contrast with hospitals with more complex patients, where the ED length-of-stay can be more than 6 h due to ED boarding. Another aspect that may explain the long ED length-of-stay and over-capacity workload is the lack of specialists available to provide definitive interventions in patients. As a result, many patients must wait several hours to be seen by specialist consultants in internal medicine, surgery, and orthopedics. As part of the response to the lack of (and need for) specialists in emergency medicine (EM), Colombians began to recognize EM as a specialty in 1996, but today, more than 15 years later, Colombia has only five EM residency programs [13, 18, 19]. Together, these programs are able to deliver a maximum of 30 EM specialists each year. Although this is an excellent start, the demand for emergency care greatly exceeds the capacity of the current residency training programs.

The NEDI-Bogotá survey demonstrates how EDs are the portal for most hospital admissions in Bogotá. Consequently, they play a fundamental role in overall hospital function and the economic status of most hospitals. Research is needed to address ED crowding and how to reduce it, including the potential role of fast-track rooms and observation units. Although these are high-impact interventions of increasing popularity in the US EDs [20], the more frequent approach in Colombia is to simply expand the number of ED beds. Cross-country research provides opportunities for more rapid adoption of effective interventions in new settings.

### Limitations

We recognize that this is an initial study with descriptive statistics, but it provides new information to guide efforts to advance emergency care in Bogotá. One limitation is that the NEDI survey is not validated. To our knowledge, a validated instrument to assess EDs worldwide does not exist. Questions from our survey were developed so that they could be used in diverse types of EDs [5]. Moreover, the survey questions have been used in several studies of US EDs [6, 20] and the actual NEDI-international instrument has been used successfully in several other countries [8–12, 14].

Another potential limitation is that this study relies on self-reported data. ED administrators were asked to supply data when available. When exact figures were unavailable, ED physicians-administrators provided their closest approximation. As the survey was anonymous, we do not suspect a systematic bias in the responses. We did note ED director uncertainty about the ED visit volume data on the original (main) survey, so we assessed the reliability of these data in a follow-up survey. By asking additional questions, we also had the opportunity to assess the internal consistency of the data and the responses were reassuring. We included the follow-up survey data for two items (annual ED visit volume, percent of ED visits that led to hospital admission), which were a little more conservative and more in line with the findings from several other NEDI-international surveys ([8–12, 14]; Table 3).

Finally, the response rate in the study was 82 %, with 15 EDs choosing not to participate. If their experience or responses differed markedly from those studied, this could introduce bias. However, the missing sites did not differ in key parameters from the >80 % who did participate in our survey. We plan to perform repeat surveys of Bogotá EDs and anticipate that the growing familiarity with our approach and sincere interest in advancing Colombian emergency care will yield higher response rates in future years.

## Conclusions

While EDs in the Colombian capital city, Bogotá, resemble US urban EDs in some ways, there are several major differences that raise questions of existing overcrowding and what will happen with the anticipated increase in ED utilization—and growing population. The NEDI-Bogotá survey provides basic descriptive information with a much larger sample than any prior study in Colombia. Like most research, it leads to further questions such as the precise reason for the high demand and long length-of-stay times. The survey also begins to describe the work environment for clinicians who provide emergency care. The generation of new data is essential to ongoing efforts to learn about emergency care models from diverse countries and, in so doing, help identify viable solutions to at least some of the problems shared by emergency physicians worldwide. At a more local level, it is important to remember that lessons from Bogotá may apply best to Bogotá. Several NEDI studies have shown how heterogeneous EDs are within single countries, and it is therefore critical to repeat similar surveys in other urban and rural regions of Colombia.

## Additional files

Additional file 1:
**Survey instrument, in English and Spanish.** The NEDI instrument includes four major branch points leading to 10 different surveys with content tailored to the specific type of ED. The major branch points are as follows: (1) contiguous vs non-contiguous, (2) triage to service vs no triage to service, (3) open 24/7 vs not open 24/7, and (4) one leader vs >1 leader. The compiled data from the surveys were subsequently combined into one dataset and recorded in Microsoft Excel. Included is a sample survey for a contiguous ED with triage to service, 24/7, and one leader. (PDF 219 kb) Additional file 2:
**Map of emergency departments in Bogotá, Colombia (** ***n*** **= 85).** The map shows the 85 EDs that were surveyed in Bogotá, Colombia. (PNG 17 kb)

## Abbreviations

CI: confidence interval
ED: emergency department
EM: emergency medicine
EMNet: Emergency Medicine Network
NEDI: National ED Inventories
US: United States.
