# Supplementary material for: Emergency department characteristics and capabilities in Bogotá, Colombia
Source: Int J Emerg Med. 2015 Aug 8;8:30. doi: 10.1186/s12245-015-0079-y (PMC4529430; doi:10.1186/s12245-015-0079-y)
Supplement: Additional file 1: — Survey instrument, in English and Spanish. The NEDI instrument includes four major branch points leading to 10 different surveys with content tailored to the specific type of ED. The major branch points are as follows: (1) contiguous vs non-contiguous, (2) triage to service vs no triage to service, (3) open 24/7 vs not open 24/7, and (4) one leader vs >1 leader. The compiled data from the surveys were subsequently combined into one dataset and recorded in Microsoft Excel. Included is a sample survey for a contiguous ED with triage to service, 24/7, and one leader. (PDF 219 kb) [file 12245_2015_79_MOESM1_ESM.pdf]

International Emergency Department Survey

The NEDI project is conducted by the Emergency Medicine Network (EMNet), an emergency medicine research group, to better understand the characteristics and scope of Emergency Departments (EDs) internationally.

Since unplanned medical problems affect people worldwide, NEDI aspires to describe emergency healthcare systems around the world. The NEDI online survey identifies similarities and differences in EDs with a philosophy that different emergency healthcare systems can learn from one another. Using technology to facilitate cooperation, we hope that NEDI will serve as a platform for exchanging ideas on improving the accessibility and quality of emergency care for all.

If you work clinically in an ED, please proceed with the survey.

Are you willing to provide descriptive information about your Emergency Department?

☐ Yes
☐ No

→ If NO, please save this survey and return to it when you are ready to fill it out. Thank you for your time.

Please answer the following questions with estimates for the year 2011 (unless otherwise indicated).

1. Is your ED physically part of a hospital (e.g. same building or adjacent):

☐ Yes ☐ No

→ If no, please specify the physical location or structure of the ED (e.g. urgent care center, mother and child center, community health center):______

2. Please estimate the total number of hospital beds; enter 999999 if unknown: _____

3. Please estimate the percent of your patients that are children; enter 999999 if unknown: _____

4. Is the physical plant of your ED:

☒ **Contiguous** – all medical (e.g. heart attack, acute asthma) and surgical (e.g. trauma) emergency care provided by a general ED in one unified area.

OR

☐ **Non-contiguous** – emergency care provided in separate areas throughout the hospital; i.e. medical emergency care (e.g. heart attack, acute asthma) provided in a geographically distinct area from surgical emergency care (e.g. trauma).

5. Is there a triage to service (i.e. triage of patients to a specific emergency service, e.g. medical vs. surgical team)?

☒ Yes ☐ No

6. Please, indicate the total number of ED beds, enter 999999 if unknown: _____

7a. In 2011, was your ED open 24 hours/day, 7 days/week?

☒ Yes ☐ No

7b. Who runs the emergency department?

☒ ED director who oversees all emergency services (e.g. medicine and surgery)
☒ Each service has its own director, however, they jointly oversee the emergency department (e.g. have board meetings).
☐ Emergency services share the same hospital area but are otherwise completely independent of one another, each with its own leadership.
☒ Other, please specify: _____

8. Please indicate whether the following types of emergencies can be treated in your ED and whether treatment is available 24/7:

	No	Yes	Not Available 24/7	Available 24/7
a. Medical- Cardiology (e.g., arrhythmia, acute myocardial infarction)	☐	☐	☐	☐
b. Medical- Oncology (e.g., fever and neutropenia)	☐	☐	☐	☐
c. Medical- Other (e.g., urinary tract infection, acute asthma)	☐	☐	☐	☐
d. Trauma (e.g., motor vehicle crash, gun shot wound)	☐	☐	☐	☐
e. Neurological and neurosurgical (e.g., acute thromboembolic stroke, intracranial hemorrhage)	☐	☐	☐	☐
f. Urological (e.g., kidney stone)	☐	☐	☐	☐
g. Obstetrical (e.g., complications of pregnancy)	☐	☐	☐	☐
h. Gynecological (e.g., ruptured ovarian cyst, yeast infection)	☐	☐	☐	☐
i. Ear, Nose, Throat (e.g., severe epistaxis)	☐	☐	☐	☐
j. Ophthalmological (e.g., acute glaucoma, eye injury)	☐	☐	☐	☐
k. Toxicological (e.g., overdose, carbon monoxide poisoning)	☐	☐	☐	☐
l. Psychiatric (e.g., psychosis)	☐	☐	☐	☐
m. Dental (e.g., tooth extraction)	☐	☐	☐	☐
n. Surgical- Oral maxillofacial (e.g., jaw fractures, oral abscesses)	☐	☐	☐	☐
o. Surgical- Plastic (e.g., severe lip laceration)	☐	☐	☐	☐
p. Surgical- Hand (e.g., tendon injury)	☐	☐	☐	☐
q. Surgical- Orthopedic (e.g., long bone fractures)	☐	☐	☐	☐

r. Surgical- General (e.g., acute appendicitis, pneumothorax)	☐	☐	☐	☐

9. Please indicate the annual number of patient visits at your ED in 2011:

Children (e.g. ages 0-17) = _____ ☐ Unknown
Adults (e.g. ages 18+) = _____ ☐ Unknown
Total (Adults + Children) = _____ ☐ Unknown

If your ED uses a different cut off age to distinguish between children and adults, please specify: _____ years

10. Approximate percent of patients that arrived at your ED by ambulance:
- ☐ < 20%
- ☐ 20 – 39%
- ☐ 40 – 59%
- ☐ > 60 – 79%
- ☐ 80% or higher
- ☐ Unknown

11. Do patients require a referral from a physician, or need to arrive by ambulance, in order to receive emergency care? (i.e., with rare exceptions, patients without a referral are not seen)
☐ Yes ☐ No

12. What is the average length of stay at your ED?
- ☐ < 1 hour
- ☐ 1-6 hours
- ☐ > 6 hours

13a. Is a physician available to the ED, 24 hours/day, 7 days/week?
- ☐ Yes—A physician is physically present in the ED, 24/7
- ☐ Yes—A physician is available to the ED from within the hospital, 24/7
- ☐ No—Variable staffing (e.g., a physician is available to the ED from outside the hospital)

13b. Is at least one nurse on duty in the ED, 24 hours/day, 7 days/week?
☐ Yes ☐ No

14. Are the following consultants available <u>in-person</u> to the ED? Residents or registrars qualify. If <u>YES</u>, please indicate, on average, how long it takes the consultant to arrive and whether the consultant is available 24/7:

	No	Yes 0-29 min	Yes 30-59 min	Yes ≥60 min	Not available 24/7	Available 24/7
a. Anesthesiologist	☐	☐	☐	☐	☐	☐
b. Cardiologist	☐	☐	☐	☐	☐	☐

c. General Surgeon	☐	☐	☐	☐	☐	☐
d. Neurologist	☐	☐	☐	☐	☐	☐
e. Neurosurgeon	☐	☐	☐	☐	☐	☐
f. Obstetrician-Gynecologist	☐	☐	☐	☐	☐	☐
g. Orthopedic Surgeon	☐	☐	☐	☐	☐	☐
h. Plastic Surgeon	☐	☐	☐	☐	☐	☐
i. Psychiatrist	☐	☐	☐	☐	☐	☐

15. In 2011, how would you describe your hospital's ED?

- ☐ Under capacity
- ☐ Good balance
- ☐ At capacity
- ☐ Over capacity

16. Please respond "yes" or "no" in reference to the following questions:

	No	Yes
a. Is there a dedicated CT scanner for the ED (i.e. preferentially for ED patients)?	☐	☐
b. Is there a cardiac monitor available immediately in the ED?	☐	☐
c. Is there a mechanical ventilator available immediately in the ED?	☐	☐
d. Is there a respiratory isolation (negative pressure) room available in the ED?	☐	☐
e. Is a computer system used to collect clinical data in the ED?	☐	☐
f. Is internet access available in the clinical area of the ED?	☐	☐
g. Is there a clinical laboratory available for your ED with the capacity to perform a potassium blood test 24/7?	☐	☐

17a. Approximate percent of your hospital's total admissions (i.e. inpatient hospitalizations) that were admitted through the ED:

- ☐ < 20%
- ☐ 20 – 39%
- ☐ 40 – 59%
- ☐ 60 – 79%
- ☐ 80 or higher
- ☐ Unknown
- ☐ Question does not apply; ED is not part of the hospital

17b. Approximate percent of ED visits that led to admission (i.e. inpatient hospitalization), including patients admitted to observation unit/status (*in some facilities, a short-stay in the "observation unit" or an admission is designated as "observation status" if the length of stay is < 24 hours*):

- ☐ < 20%
- ☐ 20 – 39%
- ☐ 40 – 59%
- ☐ 60 – 79%

☐ 80 or higher
☐ Unknown
☐ Question does not apply; ED is not part of the hospital

18. Is the ED an official department within the hospital organization (e.g. separate and independent from the Departments of Medicine or Surgery)?

☐ Yes ☐ No

→ If NO, ED is part of which department (specify): _____

Please include any comments that would help us understand your survey responses. (e.g., if your ED serves a specific patient population or is a specialty ED):

Thank you for taking the time to complete this questionnaire!

Encuesta Internacional de Departamentos de Emergencias

El proyecto NEDI es conducido por la Red de Medicina de Emergencias (EMNet, grupo de investigación en Medicina de Emergencias), conformado para entender mejor las características y alcances de los Departamentos de Emergencias (DE, equivalente a Servicios de Urgencias) a nivel internacional.

Ya que los problemas médicos inesperados afectan a las personas en todos los países, NEDI pretende describir los diferentes sistemas de emergencias en salud alrededor del mundo. La encuesta NEDI en linea identifica similitudes y diferencias en los DE siguiendo como filosofía en que los sistemas de emergencias en salud puedan aprender unos de otros. Usando la tecnología para facilitar la cooperación, esperamos que NEDI sirva como una plataforma de intercambio de ideas para mejorar la accesibilidad y calidad de la atención de emergencias para todos.

Si usted trabaja como clínico en un DE, por favor continúe con la encuesta.

¿Está dispuesto a proporcionar información descriptiva sobre su Departamento de Emergencias (Urgencias)?

☐ Sí
☐ No

→ Si la respuesta es NO, por favor guarde esta encuesta y regrese a ella cuando se encuentre listo para completarla. Gracias por su tiempo.

Por favor responda las siguientes preguntas con los estimados para el año 2011 (a menos que se indique algo distinto).

1. ¿Está su DE físicamente en un hospital (por ej. En el mismo edificio o adyacente):

☐ Sí
☐ No

→ Si la respuesta es NO, por favor especifique la ubicación o estructura del DE (por ej. En un Centro Materno-Infantil, en un Centro de Salud independiente): _____

2. Especifique el número total de camas de su hospital (ingrese 999999 si se desconoce)**: _____**

3. ¿Cuál es el porcentaje de pacientes pediátricos en su servicio? (ingrese 999999 si se desconoce**)** _____

4. La planta física de su DE está:

☒ **Contigua –** toda la atención de urgencias médicas (por ej. infarto agudo al miocardio, asma agudo) y quirúrgicas (por ej. trauma) son proporcionadas por un DE general en un área unificada.

Ó

☐ **No-contigua –** la atención de urgencias es proporcionada en áreas separadas en el hospital; ej. la atención de urgencias médicas (por ej. infarto agudo al miocardio, asma agudo) es proporcionada en un área separada geográficamente de la atención de urgencias quirúrgicas

5. ¿Existe un sistema de triage por servicio? (ej. Se realiza un triage de los pacientes a un servicio específico, Cirugía vs. Medicina)

☒ Sí ☐ No

6. Por favor indique el número total de camas en el DE (ingrese 999999 si se desconoce)**: _____**

7a. En el 2011, ¿estuvo su DE abierto las 24 horas del día, 7 días a la semana?

☒ Sí ☐ No

7b. ¿Quién dirige el departamento de urgencias?

☒ Un director de urgencias supervisa todas las urgencias (ej. médicas y quirúrgicas)
☒ Cada servicio tiene su propio director, sin embargo ellos en conjunto supervisan el departamento de urgencias (ej, mediante reuniones de la junta).
☐ El servicio de urgencias comparte la misma área del hospital, pero son completamente independientes la una de la otra, cada una con su propio líder.
☒ Otro, por favor especifique: _____

8. Por favor indique si los siguientes tipos de urgencias pueden ser tratadas en su DE. También indique si el tratamiento está disponible 24/7:

	No	Sí	No Disponible 24/7	Disponible 24/7
a. Médicas – Cardiología (por ej. Arritmia, infarto agudo al miocardio)	☐	☐	☐	☐
b. Médicas – Oncología (por ej. Fiebre y neutropenia)	☐	☐	☐	☐
c. Médicas – Otros (por ej. Infección del tracto urinario, asma agudo)	☐	☐	☐	☐
d. Trauma (por ej. Accidente automovilístico, herida por arma de fuego)	☐	☐	☐	☐
e. Neurológicas y neurocirugía (por ej. Tromboembolia aguda, hemorragia intracraneal)	☐	☐	☐	☐
f. Urológicas (por ej. Urolitiasis)	☐	☐	☐	☐
g. Obstétricas (por ej. Complicaciones del embarazo)	☐	☐	☐	☐
h. Ginecológicas (por ej. Quiste ovárico perforado, candidiasis vaginal)	☐	☐	☐	☐
i. Oído, Nariz, Garganta (por ej. Epistaxis severa)	☐	☐	☐	☐
j. Oftalmológicas (por ej. Glaucoma agudo, herida de ojo)	☐	☐	☐	☐
k. Toxicológicas (por ej. sobredosis, envenenamiento por monóxido)	☐	☐	☐	☐
l. Psiquiátricas (por ej. Psicosis)	☐	☐	☐	☐
m. Dentales (por ej. Extracción de dientes)	☐	☐	☐	☐
n. Cirugía – maxilofacial oral (por ej. Fractura de mandíbula, abscesos bucales)	☐	☐	☐	☐
o. Cirugía – Plástica (por ej. Laceración severa de labios)	☐	☐	☐	☐

p. Cirugía – Mano (por ej. Herida de tendón)	☐	☐	☐	☐
q. Cirugía – Ortopédica (por ej. Fractura de huesos largos)	☐	☐	☐	☐
r. Cirugía – General (por ej. Apendicitis aguda, neumotórax)	☐	☐	☐	☐

9. Por favor indique el número anual de visitas de pacientes a su DE durante el 2011 (ingrese 999999 si se desconoce)**:**

Niños (por ej. Edades 0-17) = _____ ☐ Desconocido
\+ Adultos (por ej. Edades 18+) = _____ ☐ Desconocido
Total (Niños + Adultos) = _____ ☐ Desconocido

Si su DE usa una edad límite diferente para distinguir entre niños y adultos, por favor especifíquela: _____ años

10. Porcentaje aproximado de pacientes que llegaron al DE en ambulancia:

☐ < 20%
☐ 20 – 39%
☐ 40 – 59%
☐ 60 – 79%
☐ 80% o más
☐ Desconocido

11. ¿Requieren los pacientes ser referidos por un médico, o requieren llegar en una ambulancia, para poder recibir la atención de urgencias? (por ej. con excepciones raras, los pacientes sin una referencia no son atendidos)

☐ Sí ☐ No

12. ¿Cuál es el tiempo promedio que permanecen los pacientes en el área de su DE?

☐ < 1 hora
☐ 1-6 horas
☐ > 6 horas

13a. ¿Hay un médico disponible en el DE, 24 horas al día, 7 días a la semana?

☐ Sí—Hay un médico presente físicamente en el DE, 24/7
☐ Sí—Hay un médico disponible para el DE dentro del hospital, 24/7
☐ No—Personal variable (por ej. un médico está disponible para el DE fuera del hospital)

13b. ¿Hay por lo menos una enfermera de turno en el DE, 24 horas al día, 7 días a la semana?

☐ Sí ☐ No

14. ¿Están los siguientes especialistas para interconsulta disponibles <u>en persona</u> para el DE? Los residentes o médicos en formación califican. **Si la respuesta es SI, por favor indique, en promedio, cuánto tiempo le toma al interconsultante llegar y si éste está disponible 24/7:**

	No	Sí 0-29	Sí 30-59	Sí ≥60	No Disponible	Disponible 24/7

		min	min	min	24/7	
a. Anestesiólogo	☐	☐	☐	☐	☐	☐
b. Cardiólogo	☐	☐	☐	☐	☐	☐
c. Cirujano General	☐	☐	☐	☐	☐	☐
d. Neurólogo	☐	☐	☐	☐	☐	☐
e. Neurocirujano	☐	☐	☐	☐	☐	☐
f. Obstetra-Ginecólogo	☐	☐	☐	☐	☐	☐
g. Cirujano Ortopédico	☐	☐	☐	☐	☐	☐
h. Cirujano Plástico	☐	☐	☐	☐	☐	☐
i. Psiquiatra	☐	☐	☐	☐	☐	☐

15. En el 2011, ¿cómo describiría el DE del hospital?

☐ Por debajo de la capacidad
☐ Buen balance
☐ En la capacidad
☐ Por encima de la capacidad

16. Por favor responda "sí" o "no" a las siguientes preguntas:

	No	Sí
a. ¿Hay un Tomógrafo dedicado al DE? (disponible preferencialmente para los pacientes del DE)	☐	☐
b. ¿Hay un monitor cardiaco disponible inmediatamente en el DE?	☐	☐
c. ¿Hay un respirador mecánico disponible inmediatamente en el DE?	☐	☐
d. ¿Hay una sala de aislamiento respiratorio (presión negativa) disponible en el DE?	☐	☐
e. ¿Es utilizado un sistema computarizado para recoger datos clínicos en el DE?	☐	☐
f. ¿Existe acceso a internet en el área clínica de su DE?	☐	☐
g. ¿Existe un laboratorio de análisis clínicos disponible para su DE con la capacidad de medir niveles de Potasio 24/7?	☐	☐

17a. Porcentaje aproximado del total de admisiones del hospital (por ej. Hospitalizaciones) que fueron admitidas a través del DE:

☐ < 20%
☐ 20 – 39%
☐ 40 – 59%
☐ 60 – 79%
☐ 80% o más
☐ Desconocido
☐ No se aplica; el DE no es parte de un hospital

17b. Porcentaje aproximado de visitas de emergencia al DE que terminan con admisión al hospital (por ej. hospitalización) incluyendo pacientes ingresados a la unidad de observación o en estado de observación (*En algunas instalaciones, una estadía corta en la "unidad de observación" o una admisión es clasificada como "estado de observación" si la estadía es menor a 24 horas):*

☐ < 20%
☐ 20 – 39%
☐ 40 – 59%
☐ 60 – 79%
☐ 80% o más
☐ Desconocido
☐ No se aplica; el DE no es parte de un hospital

18. ¿Es el DE un departamento oficial dentro de la organización del hospital (por ej. independiente y separado de los Departamentos de Medicina o Cirugía)?

☐ Sí ☐ No

→ Si la respuesta es NO, el DE es parte de qué departamento(s) (especifique): _____

Por favor indique algún comentario que nos podría ayudar a entender las respuestas de su encuesta. (por ej. Si su DE atiende a una población específica de pacientes o es un DE de especialidad):

¡Gracias por tomar el tiempo para completar este cuestionario!
