# Supplementary figures and images for: Emergency department characteristics and capabilities in Bogotá, Colombia
Source: Int J Emerg Med. 2015 Aug 8;8:30. doi: 10.1186/s12245-015-0079-y (PMC4529430; doi:10.1186/s12245-015-0079-y)

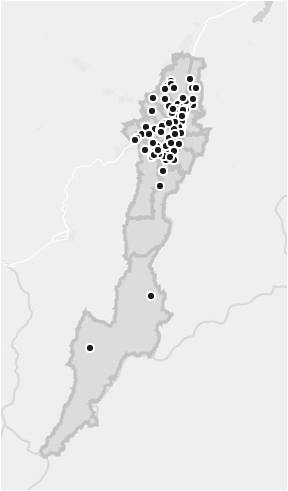

Supplement: Additional file 2: — Map of emergency departments in Bogotá, Colombia ( n = 85). The map shows the 85 EDs that were surveyed in Bogotá, Colombia. (PNG 17 kb) [file 12245_2015_79_MOESM2_ESM.png]
